# Modelling the role of glucocorticoid receptor as mediator of endocrine responses to environmental challenge

**DOI:** 10.1098/rstb.2022.0501

**Published:** 2024-03-25

**Authors:** Blanca Jimeno, Juan G. Rubalcaba

**Affiliations:** ^1^ Instituto Pirenaico de Ecologia (IPE), CSIC, Avda. Nuestra Señora de la Victoria 16, 22700, Jaca, Spain; ^2^ Departamento de Biodiversidad, Ecología y Evolución, Facultad CC Biológicas, Universidad Complutense de Madrid, José Antonio Nováis 12, 28040, Madrid, Spain

**Keywords:** stress response, endocrine flexibility, stress resilience, dynamic model, glucocorticoid, hypothalamic–pituitary–adrenal (HPA) axis

## Abstract

Glucocorticoid hormones (GCs) modulate acute ‘stress’ responses in vertebrates, exerting their actions across many physiological systems to help the organism face and overcome challenges. These actions take place via binding to the glucocorticoid receptor (GR), which determines not only the magnitude of the GC-mediated physiological response but also the negative feedback that downregulates GCs to restore homeostasis. Although GR function is assumed to determine GC regulation capacity, the associations between GR abundance and individuals' coping abilities remain cryptic. We developed a dynamic model fitted to empirical data to predict the effects of GR abundance on both plasma GC response patterns and the magnitude of GC-mediated physiological response. Individuals with higher GRs showed lower GC exposure, stronger physiological responses and greater capacity to adjust this response according to stressor intensity, which may be translated into more resilient and flexible GC phenotypes. Our results also show that among-individual variability in GR abundance challenges the detectability of the association between plasma GC measurements and physiological responses. Our approach provides mechanistic insights into the role of GRs in plasma GC measurements and function, which point at GR abundance fundamentally driving complex features of the GC regulation system in the face of environmental change.

This article is part of the theme issue ‘Endocrine responses to environmental variation: conceptual approaches and recent developments’.

## 1. Introduction

Understanding the factors driving differences in organismal responses to internal and external perturbations is fundamental towards predicting their capacity to cope with environmental change. Glucocorticoid hormones (GCs) have been widely measured in the field of conservation and ecophysiology as indicators of animal welfare and physiological status in vertebrates [1–4], and generally assumed to be directly or indirectly related to fitness prospects and coping abilities [5–7]. This interpretation partly originates from the acute increase in plasma GCs that animals show in response to sudden, unpredicted stimuli (‘stressors’) that pose a threat to homeostasis. This response pattern, along with the deleterious effects associated GC exposure [8,9], has led to the frequent assumption that higher GC levels are a proxy of poorer physiological state (reviewed in [7,10]). This premise contrasts with the existing evidence of the adaptive role of GC increases (including acute responses) under certain environmental contexts, as they allow the organisms to adjust their physiology to prevailing demands ([8,11]; see below). Indeed, studies testing for correlations between plasma GC concentrations (both baseline and stress-induced) and fitness components have yielded contrasting results [4,7,12–14]. In this context, recent works have claimed that a negative association between GCs and physiological state is an overly simplistic interpretation of the complexity of GC function, claiming the need to study them from a wider, more integrative perspective [15–19]. In the meantime, the mechanistic underpinnings underlying variation in GC responses remain poorly understood.

GC function entails complex cascade effects involving several physiological systems and the transcription of multiple genes [8,20–23], which help the organism to prepare for, face, or recover from environmental challenges [8,18,23]. Because these actions only take place once GCs bind to receptors, researchers have long identified the interest of targeting receptor function when investigating GC variation (e.g. [18,24–26]). Indeed, recent studies have questioned the utility of interpreting plasma GC concentrations alone, highlighting the need to focus on key regulatory components involved in GC regulation and activation of GC-mediated physiological functions [18,25–31]. This change of perspective has led to researchers proposing new integrative measurements that aim to capture the complexity of the system and its emerging properties, with the hypothesis that these traits may ultimately connect individual phenotypes to performance and fitness. Examples of these complex traits are the ability of the organism to mount an acute response and efficiently return to baseline levels via negative feedback [11,32–34], or the capacity of an individual to adjust such response patterns to the environmental context (i.e. ‘endocrine flexibility’; [35–37]). However, in order to understand these emerging properties and their role in determining animal coping abilities, we need to identify the factors mediating GC function, focusing on the ´integration steṕ (i.e. incorporation into target tissues; [31]). According to this framework, key regulatory components such as hormone receptors fundamentally determine endocrine responses and coping abilities within fluctuating environments. Yet, due to the complex nature of GC regulatory dynamics (e.g. [38,39]) and to the methodological limitations associated with receptor measurements (i.e. the impossibility of obtaining repeated samples from the same individuals unless from peripheral tissues; see below), the associations between GC regulatory components, concentrations and physiological functions are difficult to predict. This becomes particularly evident in wild populations in which individuals differ in their capacity to cope with environmental challenges, and in which repeated or invasive sampling is often difficult. Predicting these associations in nature thus requires a deep understanding of the GC regulation system, from GC synthesis to integration into target tissues (e.g. [11,31,32,40]).

GCs are produced in the adrenal gland and released into the blood following a cascade of reactions in the hypothalamus and pituitary gland (i.e. hypothalamic–pituitary–adrenal (HPA) axis; [8]). They circulate towards target tissues and cells, where the amount of GCs available to receptors will be determined by corticosteroid-binding globulines (CBG) and other binding proteins [41]. GCs exert physiological actions by binding to two types of nuclear receptors: the mineralocorticoid receptor (MR) and the glucocorticoid receptor (GR) [8,9,18,21,24]. MRs are expressed in specific tissues (e.g. hippocampus, liver, kidney, heart, colon), while GRs are expressed in nearly every cell type, including nucleated blood cells [9,42–44]. Because the MR has a higher affinity for GCs than the GR, it is saturated at lower circulating concentrations, mainly regulating traits associated with metabolism, foraging and activity level, but also mediating fast nongenomic actions and showing lower affinity—in the range of the GR—when residing in the membrane [45]. GRs are mainly recruited with increasing level of GCs and thus they regulate activities associated with acute GC responses [21,46]. The role of GRs in GC function and regulation is two-fold: on the one hand, activation of GRs by GCs at daily peaks and with acute (e.g. ‘stress-induced’) responses results in widespread physiological effects [8,47,48] that culminate in phenotypic adjustments in metabolism (i.e. glucose synthesis and mobilization; see below), immune system and central nervous system function, or behaviour [49]. On the other hand, GC–GR binding within HPA tissues in the brain (i.e. hypothalamus and pituitary) mediates the negative feedback that downregulates HPA axis activity by reducing the production of GCs down to basal levels and restoring homeostasis [8,11,33,34]. Therefore, the number of GRs in central tissues should simultaneously determine the amount of GCs integrated via receptor binding, the magnitude of the GC-mediated physiological response and the intensity of the negative feedback that reduces GC concentration down to baseline levels. In contrast to the biomedical literature, where the central role of GR on GC regulation is relatively well established [20–22], the generality of these associations remains unknown in free-living populations. Indeed, several ecological studies have pointed towards focusing on GR function (e.g. [18,21,25,29–31]) and its regulatory components (e.g. FKBP5; [40]) as a key step towards interpreting and predicting GC variation and obtaining information on coping abilities.

To investigate how GRs modulate both GC levels and GC-mediated physiological responses and predict the physiological consequences of varying GR abundance, we need to simulate the overall HPA axis dynamics while accounting for reaction kinetics governing GC–GR bindings. Endocrine dynamic models provide a means to calculate the concentrations of different interacting hormones and their receptors and predict how hormones respond to changes in regulatory components, like receptor levels (e.g. [38,50,51]). These models thus help us to understand and infer the mechanistic associations among regulatory components within endocrine systems, the associated responses and the relative importance that certain regulatory components have in environmental coping and phenotypic adaptation. In this work, we develop a dynamic model to predict how changes in GR abundance within the HPA axis simultaneously affects (i) plasma GC acute response patterns (i.e. the dynamics of circulating GCs during an acute stress response) and (ii) the magnitude of GC-mediated physiological response (i.e. cascade effects triggered by GC binding to receptors). We first fitted the model to empirical plasma GC measurements including different steps of the acute response: baseline levels, stress-induced levels, and GC concentrations after dexamethasone (i.e. feedback efficiency). Then we tested how these different measurements might vary among individuals with different GR abundances and under different stressor intensities. We also investigated how differences in GR abundance affects the magnitude of the GC-mediated physiological response of individuals to these challenges, and how this effect may shape the detectability of an association between plasma GCs and GC-mediated physiological responses in wild populations.

## 2. Methods

### (a) Dynamic HPA axis model

**Figure 1.**
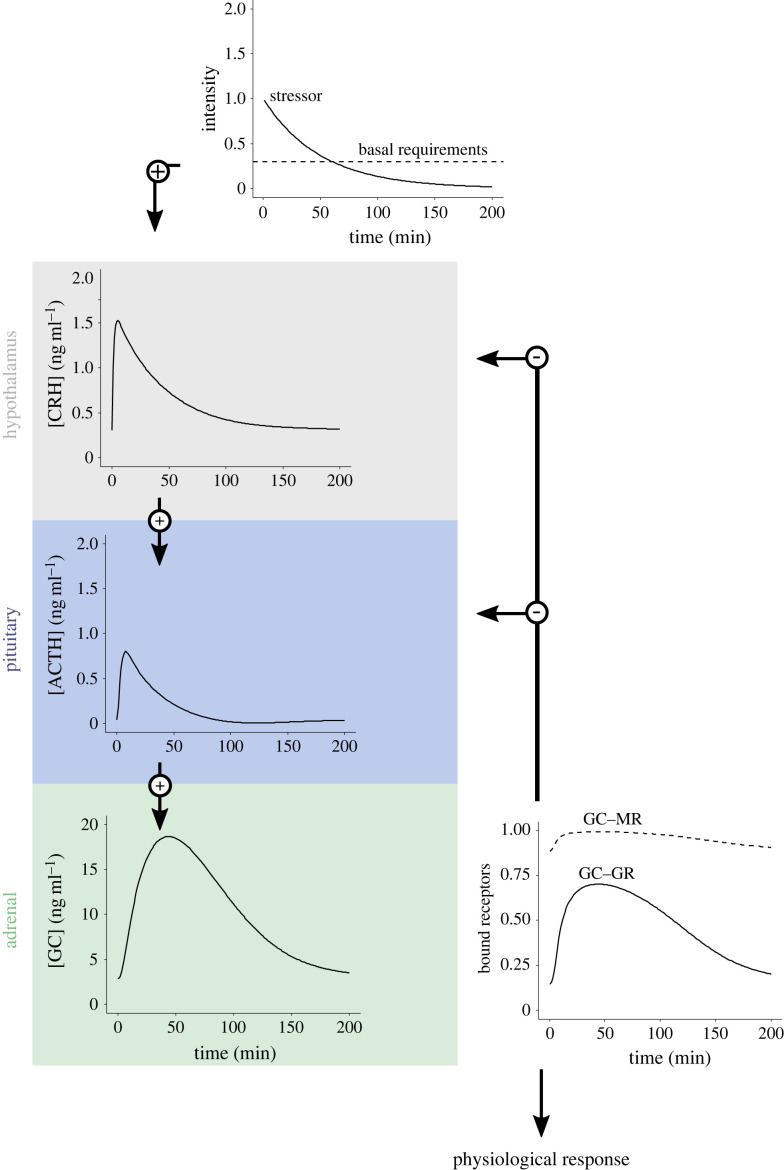
Diagram of the dynamic HPA-axis model describing the effect of external stressor and basal metabolic requirements on the concentration of CRH, ACTH and glucocorticoids (GCs). GCs bind to the glucocorticoid receptors (GR) and mineralocorticoid receptor (MR), triggering the negative feedback that inhibits GC production. Simultaneously, bound GR activate the physiological response to the initial stressor.

We developed a model of the hormone synthesis dynamics at the HPA axis to investigate the variation in plasma glucocorticoid responses (GCs) to an external stimulus, and how the magnitude of these responses varies due to changes in the abundance of glucocorticoid receptors (GRs) in HPA tissues. The model simulates an initial input (acute stressor) that activates the cascade of hormone synthesis by the hypothalamus (corticotropin releasing hormone, CRH), pituitary (adrenocorticotropic hormone, ACTH) and adrenals (glucocorticoids, GCs; figure 1). GCs then bind to both mineralocorticoid (MRs) and glucocorticoid receptors (GRs) of the hypothalamus and pituitary activating the negative feedback that downregulates GC concentrations (figure 1). Here, we assume that both MRs and GRs activate a negative feedback. Note that dynamic effects of MRs remain elusive but empirical evidence suggests that MRs dynamically regulate HPA activity via feedback mechanisms ([52–55], but see [56]).

The stimulus that activates the HPA axis was simulated as the sum of an acute stressor and basal energetic requirements. The acute stressor was modelled as an exponential decay in relation to time [51] and the basal requirements as a constant (B) representing the minimum energy demand and baseline function of the HPA axis:

2.1 $$p(t,b,\alpha )=B+b\alpha e^{-\alpha t}$$

where parameter *b* modulates the intensity of the stressor (i.e. the magnitude of the effect of the external stressor on the HPA axis), which may be related to the increase in metabolic demand experienced by the organism [19]. This stimulus (equation (2.1)) triggers the synthesis of CRH (*C*) in the hypothalamus (equation (2.2)), then *C* stimulates the production of ACTH (*A*) in the pituitary (equation (2.3)), and finally, *A* activates the synthesis of steroids (i.e. GCs) (*O*) in the adrenals (equation (2.4)):

2.2 $$\frac{dC}{dt}=p(t)-b_{C}C-k_{\mathrm{CG}}O_{G}-k_{\mathrm{CM}}O_{M}$$

2.3 $$\frac{dA}{dt}=k_{A}C-b_{A}A-k_{\mathrm{AG}}O_{G}-k_{\mathrm{AM}}O_{M}$$

2.4 $$\frac{dO}{dt}=k_{O}A-b_{O}O+k_{-M}O_{M}+k_{-G}O_{G}-k_{M}O_{M}-k_{G}O_{G}$$

where *−b_C_ C*, *−b_A_A*, and *−b_O_O* model constant degradation rates of CRH, ACTH and GCs, and *k*_A_*C* and *k_O_A* are production rates of ACTH and GCs, respectively. The terms *O*_G_ and *O*_M_ represent GCs associated with either GR or MR that are responsible for the negative feedback of the HPA system. Thus, the constants k_CG_ and k_CM_ modulate the strength of the negative feedback induced by the ligand–receptor complexes GC–GR and GC–MR in the hypothalamus; and k_AG_ and k_AM_ are their equivalent in the pituitary. For simplicity, we assumed that the strength of the negative feedback is equal for both GR and MR at the hypothalamus and pituitary (k_CG_ = k_CM_ = k_AG_ = k_AM_ set here to 0.1) and thus differences among receptors are entirely determined by their initial abundance and affinity for GCs.

GCs and the receptors GR and MR form ligand–receptor complexes *O*_G_ and *O*_M_, respectively, with rates of reaction denoted by *k*_G_, *k_–_*_G_, *k*_M_, *k*_–__M_, so that the reaction equations can be written as:

2.5 $$O+G\overset{K_{G}}{\underset{K_{-G}}{⇌}}O_{G}$$

2.6 $$O+M\overset{K_{M}}{\underset{K_{-M}}{⇌}}O_{M}$$

Hence, the dynamic of the *O*_G_ and *O*_M_ complexes and free receptors G and M are:

2.7 $$\frac{dO_{G}}{dt}=k_{G}OG-k_{-G}O_{G}$$

2.8 $$\frac{dG}{dt}=k_{-G}O_{G}-k_{G}OG$$

2.9 $$\frac{dO_{M}}{dt}=k_{M}OM-k_{-M}O_{M}$$

2.10 $$\frac{dM}{dt}=k_{-M}O_{M}-k_{M}OM$$

In chemical equilibrium, $K_{G}=(k_{-G}/k_{G})$ and $K_{M}=(k_{-M}/k_{M})$, the concentrations of *O*_G_ and *O*_M_ binding complexes are given by:

2.11 $$O_{G}=\frac{OG}{O+K_{G}}$$

2.12 $$O_{M}=\frac{OM}{O+K_{M}}$$

where *K*_G_ and *K*_M_, are the Michaelis constants of GR and MR, respectively, representing the affinity of the receptors to GC molecules. Equation (2.11) gives the abundance of GCs bound to the GRs within the HPA axis, responsible for triggering GC-mediated physiological responses in these tissues. Thus, we used this equation to model the magnitude of the physiological response as a result of changes in the abundance of GR (i.e. by setting the initial value of free GR, parameter *G*) and in response to different levels of the stressor (parameter *b*, equation (2.1)).

### (b) Fitting the model

Although the model's parameters represent measurable biochemical rates, we lack experimental measurements of most of these parameters and do not know the extent to which their values differ among individuals in natural populations. Hence, instead of using experimentally measured parameter values, we fit the model statistically to already published empirical data on plasma GC levels of three bird species: zebra finch (*Taeniopygia guttata*; [58,60]), house sparrow (*Passer domesticus*; [29]) and tree swallow (*Tachycineta bicolor*; [30]). These studies measured GR expression in central and peripheral tissues, capturing natural variation in GR among individuals from both captive [59] and wild [29,30] populations. Empirical GC responses (i.e. GC profiles) included standardized baseline (i.e. sampled within 3 min after disturbance) and stress-induced levels (i.e. sampled at min. 20–30 after disturbance and in response to a standardized stressor), and also GC concentrations measured after dexamethasone administration (i.e. DEX, sampled at min. 60–80), a GC analogue used to determine the maximum capacity of the HPA axis to downregulate GC levels via negative feedback (e.g. [11,16,33]). In the zebra finch study, GC concentrations were also measured after adrenocorticotropic hormone administration (i.e. ACTH, sampled at min. 100), GC precursor used to determine the maximum GC production capacity; e.g. [57]).

To fit the model to empirical data, we adjusted the values of the parameters for CRH degradation rate (*b*_C_), ACTH production (*k*_A_) and degradation rate (*b*_A_), GC production (*k*_O_) and degradation rate (*b*_O_), the binding and releasing constants of GR and MR (*k*_G_, *k_–_*_G_, *k*_M_, *k_–_*_M_), and the initial levels of GR and MR (*G* and *M* for *t* = 0). Note that the estimated parameter values might not necessarily capture the actual biochemical rates but allowed the model to reproduce realistic GC profiles in modelled populations. We assigned starting values for these parameters by visually fitting the model to the observed GC concentrations at each sampling time. Then, we maximized the log-likelihood function using the Nelder-Mead method in the general-purpose optimization function in R 4.2.2 [60]. To model ACTH injections, we simulated an increase in ACTH at minute 80, mimicking the experimental trials.

### (c) Predicting GC profiles and physiological responses

We are interested in predicting how GC concentrations and stress-induced physiological responses (i.e. concentration of GC–GR binding complexes, equation (2.11)) vary among individuals with different GR abundance. Using the fitted model, we changed the value of the GR parameter—keeping the rest of the parameters at their estimated values—and computed the resulting change in baseline, stress-induced, post-DEX and post-ACTH GC levels. By doing this we generated predicted relationships between GC concentrations at each time-point and GR abundance. Then, we compared the predicted associations with the observed among-individual relationships between plasma GC concentrations and relative GR expression reported in the above-mentioned studies.

We also examined the association between GC concentration at the different time-points and stress-induced physiological responses across levels of GR abundance and for different intensities of the input stressor (parameter *b* in equation (2.1)). Specifically, we modelled GC profiles and physiological responses of individuals with different GR abundances in response to different stressors, and tested how GR abundance affects the range of GC concentrations and physiological responses that individuals can show in response to a variety of stressors.

Finally, we are interested in understanding how changing GR abundance affects the expected relationship between physiological response and plasma GC concentration. GR abundance in HPA tissues modulates both GC concentrations (by affecting the negative feedback on GC production) and the magnitude of the stress-induced physiological response. Therefore, although physiological response is positively correlated with GC level (following a saturation curve, equation (2.11)), variation in GR abundance among individuals could affect the observed relationship between physiological response and GC levels. We modelled physiological response saturation curves in relation to the level of GC for different GR abundances and explored the expected association between physiological response and plasma GC concentration at different GC sampling times. Note that the predictions derived from our model are restricted to GR variation within HPA tissues, but similar patterns in the associations between GR abundance and GC-mediated physiological responses should be expected when GR variation in central tissues reflects such variation in peripheral ones (see §4).

## 3. Results

### (a) Model fitting

**Figure 2.**
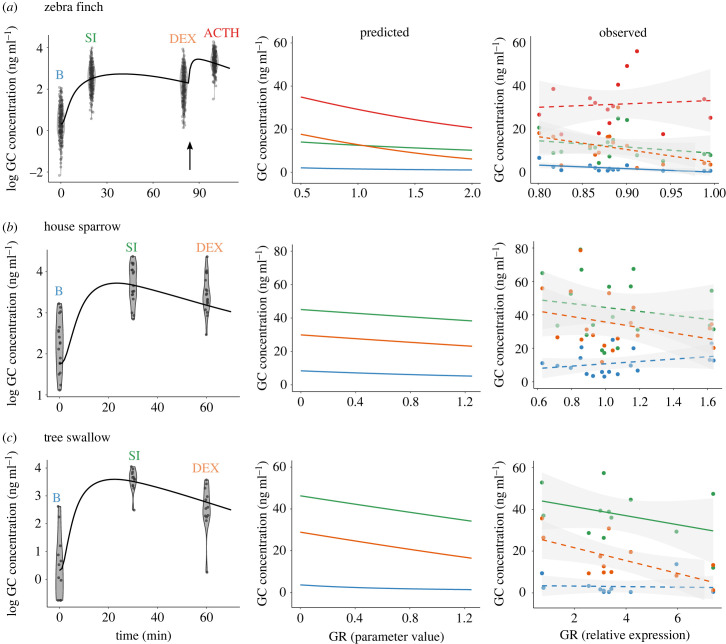
Model fit (solid lines) to empirical glucocorticoid data (points) including baseline (B) and stress-induced levels (SI), and GC concentrations after dexamethasone (DEX) or ACTH injections (the arrow in (*a*) indicates the time at which ACTH was injected). We examined how GC concentrations at each level change in relation to the level of glucocorticoid receptor (GR) using both the model (predicted) and empirical data (observed). Empirical data from Jimeno *et al.* [57,59] (*a*), Zimmer *et al.* [29] (*b*) and Zimmer *et al.* [30] (*c*).

Using the model fitted to empirical GC data across bird species (see electronic supplementary material, table S1), we generated predictions on how GC concentrations at either baseline, stress-induced, post-DEX and post-ACTH levels change as a function of GR abundance. In the model, increasing GR reduced GC concentrations at all timepoints and in all three species, although the slope of the association was stronger at higher GC concentrations being almost absent at low GC concentrations (i.e. after-ACTH > stress-induced > after-DEX > baseline; figure 2). Although observed relationships between GC and GR expression show high residual variation, making it difficult to identify empirical patterns, the observed relationships generally met the model predictions with most of the associations between GC and GR expression being negative or nonsignificant (figure 2).

### (b) GC profiles and physiological responses

**Figure 3.**
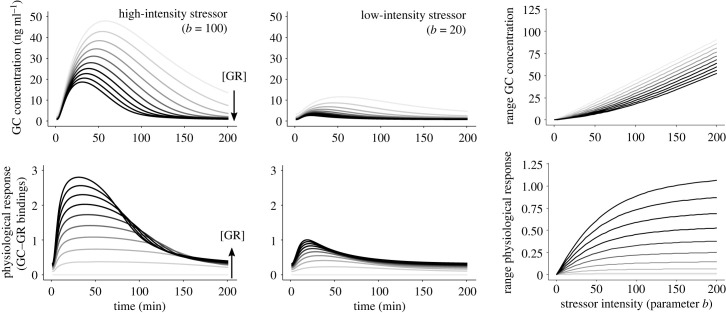
Predicted glucocorticoid (GC) profiles and GC-mediated physiological response (GC–GR bindings) for increasing concentrations of glucocorticoid receptor (GR, greyscale) and across levels of stressor intensity (parameter *b* in equation (2.1)).

Individuals showing different GR abundance within the HPA axis are predicted to respond differently to the input stressor, in terms of both changes in plasma GC concentrations and magnitude of stress-induced physiological response. First, plasma GC concentrations are higher when GRs are lower, this effect being more evident at the peak of the GC response (figure 3). This pattern occurs because GR abundance directly influences the effect of the negative feedback that downregulates GC production. At low GC concentrations most GRs are still unbound and hence differences in GR abundance have lower influence on GC concentration. At the peak of the GC response, GCs saturate GRs, especially in individuals with lower GR abundance, which rapidly reduces the effectiveness of the negative feedback, facilitating the increase in GC concentration and hampering their return to baseline levels. Interestingly, a higher abundance of GR slightly reduces the rate of stress-induced GC increase in plasma and accelerates the rate of decrease. As a result, the time at which GCs reach the peak concentrations occurs later, and GC concentrations remain elevated for longer when GR abundance is low (figure 3).

Second, GR abundance influences the magnitude of the GC-mediated physiological response to the stressor, an effect that is also shaped by stressor intensity. Under a high-intensity stressor, the physiological response reaches an asymptote caused by the saturation of GRs. Individuals with low GR reach this asymptotic response earlier, showing weaker and longer-lasting stress-induced physiological responses (figure 3). By contrast, individuals with higher GR display a stronger physiological response and decrease it faster as GCs return to basal levels (figure 3). Reducing the stressor intensity reduces the peak GC concentration and thus the range of both GC concentrations and physiological responses (i.e. maximum – minimum levels across the stress response; figure 3). Individuals with low GR display higher ranges in GC concentration but lower ranges in the magnitude of their physiological response. More importantly, the range of physiological response displays a saturation curve with increasing stressor intensity. As a result of this saturation, individuals with low GR display similar physiological responses to either intermediate or high-intensity stressors. That is, the sensitivity of the physiological response to changes in stressor intensity is lower in individuals with low GR. By contrast, individuals with high GR exhibit greater sensitivity even at relatively high stressor intensities, suggesting that they have greater capacity to accommodate their physiological response to different levels of the stressor.

**Figure 4.**
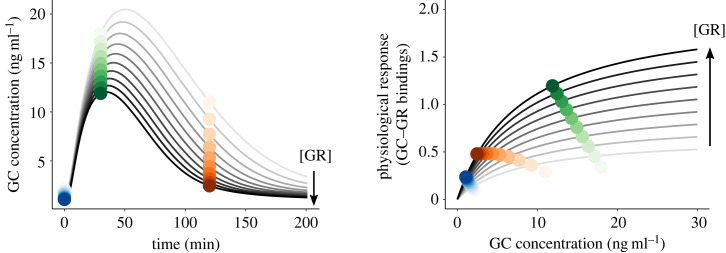
Predicted glucocorticoid (GC) and physiological response (GC–GR bounds) for increasing concentrations of glucocorticoid receptor (GR). Coloured circles represent GC samples taken at either baseline (*t* = 0 min, blue), stress-induced (30 min, green), or recovery (120 min, orange).

We finally simulated GC levels and physiological responses at three sampling times (*t* = 0, baseline; 30 min, stress-induced; and 120 min recovery levels) for variable GR abundance to examine what is the expected relationship between physiological response and plasma GC concentration. As previously outlined, low GR abundance is associated with higher GC concentrations and weaker physiological response. Therefore, although physiological response is positively correlated with GC level (following a saturation curve), variation in GR abundance could mask this relationship or even transform it into an artefactual negative trend if the comparison is tested using GC samples measured at one time-point only (figure 4).

## 4. Discussion

We developed a dynamic HPA axis model to investigate the role of GR abundance in standardized plasma GC measurements and the magnitude of the expected GC-mediated physiological responses in a context of acute endocrine responses. Our model, fitted to empirical data, provides quantitative predictions for the negative effect of GR abundance on plasma GCs and the positive effect on the GC-mediated physiological responses, whose magnitude is shaped by stressor intensity. Results also show that variability in GR abundance among individuals leads to complex associations between single plasma GC measurements and physiological responses, due to the two-fold influence of GR on GC-induced negative feedback and GC-mediated physiological response. Model outcomes thus point at GR abundance fundamentally driving complex features of GC regulation, such as stress resilience and endocrine flexibility.

In our model, increasing GR abundance in HPA tissues (hypothalamus and pituitary) reduces plasma GC levels, an effect that was stronger at higher GC concentrations (i.e. stress-induced). A negative association between GR expression and stress-induced GCs has previously been reported in captive mammals [22] and free-living birds ([61], but see [30]). Other bird studies have reported positive associations between the magnitude of the stress response and GR expression in HPA axis tissues [30] and in blood [59]. Our model also predicts that higher GR abundance slows down the acute increase in GCs, and accelerates the return to baseline concentrations. Hence, the model predicts that individuals with fewer GRs will require more time to mount an endocrine response to the stressor, maintaining higher plasma GC concentrations for longer periods and thus showing lower feedback efficacy. Negative feedback is thought to be crucial to cope with environmental challenges [33,34,46,62], and indeed previous biomedical research has reported positive associations between GR expression and feedback efficacy in humans [46,63–65]. In the field of ecology, a few recent examples in bird species also report positive associations between GR expression in HPA tissues and enhanced negative feedback responses, in line with our results [29,30,66]. From an ecological perspective, our results would imply that individuals with fewer GRs are exposed to larger amounts of GCs, potentially suffering higher physiological costs derived from the downstream effects of very high or long-lasting plasma GC levels. The above predictions also suggest that standardized GC measurements at certain time points (e.g. 0 min, 30 min, 60 min), as they are most often quantified [16,67–69], may fail to capture the peak in GCs across individuals differing in GR abundance, minimizing or masking among-individual differences in stress-induced responses if taken too early [31]. The above results add-up to previous literature questioning the ecological interpretation of single plasma GC measurements, advocating for more complex and integrative measurements and suggesting new traits to measure in order to capture the complexity of GC regulation (i.e. stress resilience, HPA flexibility; [35–37,70]. We note however, that these measurements may fail to capture GC functional effects if only focusing on plasma measurements, and that taking multiple within-individual measurements is logistically complex. Alternatives may imply focusing on more complex traits that capture or mediate the binding stage, which eventually determines GC actions, such as GR function or its regulation by FKBP5 [31,40,71]. Quantifying these traits, however, also entails important limitations, such as the difficulties associated with obtaining repeated within-individual measurements across tissues unless working on peripheral ones (e.g. blood; [31]), which may pose a challenge to testing our predictions. When this is the case, our model may be used to infer the extent to which variation in the magnitude of plasma GC responses within a population may be attributed to differences in GR levels: the model could be fitted to the observed individual GC profiles, leaving GR abundance as a free parameter. This type of analysis could be used as a quantitative basis to decide over the need or interest of performing further GR measurements. We encourage researchers to direct their efforts towards overcoming the methodological difficulties that measuring integrative traits entail, as well as delineating the limitations derived from their interpretation, for instance by testing whether environmentally induced variation in peripheral tissues reflects variation in central or regulatory ones.

The model predicted an effect of stressor intensity on the association between GR abundance and GC-mediated physiological response, with individuals exhibiting higher GR levels showing physiological responses of greater magnitude and higher sensitivity to changes in stressor intensity. From an ecological point of view, this would imply individuals with higher GR abundance showing stronger GC-mediated physiological responses, which along with the lower GC exposure may be translated into more efficient responses to the stressors. Whether this association holds at an organismal level (i.e. across different tissues) is subject to the extent to which GR abundance or its variation covaries among tissues within an individual (see below). This projection highly concurs with a recent work predicting that individuals with higher GR abundance will show a faster stress-induced response in plasma GCs and higher magnitude of the physiological GC effects, and will be sensitive to a wider range of circulating GCs [31]. In the light of these predictions, individuals with higher GR abundance would have a greater capacity to adjust phenotypically to prevailing conditions by showing a greater range of achievable phenotypes [31], a characteristic known as endocrine flexibility and suggested to be linked to variation in receptor affinity [35].

We found an inconsistency in the association between GC-mediated physiological response and plasma GCs measured in a standardized way (e.g. one or two GC measurements at specific times after disturbance) in populations exhibiting variation in GR abundance. According to the model, the detected associations between single plasma GC measurements and GC-mediated physiological responses could range from positive to negative, including lack of association. This finding could explain the contrasted results reported by studies investigating the association between GC cascade effects or functional consequences and plasma GCs [72,73]. Indeed, recent work suggests that researchers should rather focus on the associations between GC-mediated physiological response and HPA regulatory components mediating GC integration into tissues (e.g. GR; [31,40]). Note, however, that other additional components not included in our model may play a role in the integration of GC into tissues—e.g. by tuning GR function—potentially also showing an association with HPA-mediated physiological response (see below). Because measuring GR abundance in central tissues entails methodological constraints, particularly in wild populations, GC concentrations may be used to gather information on the physiological response when including multiple within-individual samples throughout the acute response. Alternatively, measuring GR expression in peripheral tissues (i.e. blood) rises as a promising research path to obtain information on GC regulation and organismal responses to challenge. However, further research evidence is required to support this assumption, as the extent to which GR expression in blood reflects GR expression in central tissues remains debated [27,31,74].

Our modelling approach allowed us to provide mechanistic insights on the role of HPA-axis regulatory components in determining individual responses to environmental stressors. This approach, however, has limitations. First, although we restricted our predictions to GR variation, we acknowledge the complexity of the system, which includes several regulatory components known to mediate pre-binding GC availability (e.g. CBG, 11β hydroxysteroid dehydrogenase [25,26,28,40,75,76] or receptor affinity (e.g. FKBP5 [35,40]) as well as post-transcription modulators, which were not included in the model. Likewise, we acknowledge that recent evidence suggests additional mechanisms regulating GC production (e.g. self-inhibitory feedback within the adrenals [77]; or the role of the hippocampus on HPA regulation dynamics [78]). Variation in these and other components might shape or mask the predicted associations, and thus further research is needed to test the generality of our predictions. Second, we analyse variation in one type of glucocorticoid receptor only (i.e. GR) because we focused on short time periods and acute responses, but there is evidence suggesting an effect of MR levels, or MR/GR ratio, on GC functional outcomes [21,79,80]. Third, we focused on a relatively simple relationship to simulate GC-mediated physiological responses. However, these responses are complex and may be subjected to additional regulatory components with additional interactions and nonlinear effects (e.g. step functions in response to GC increase) that could mask the associations predicted by the model. Fourth, we expect our predictions to be subjected to additional variation due to within-individual differences in receptor abundance by tissue, leading to tissue-specific receptor sensitivity to GCs [27,31,71].

From an ecological perspective, our results suggest that higher GR levels are beneficial, for three reasons. First, higher GR abundance implies lower GC exposure during acute responses and faster return to baseline levels. Second, higher GR abundance allows individuals to adjust the response to variation in stressor intensity, and thus to a wider range of fluctuations in energy demand [19]. Third, higher GR abundance allows for GC-mediated physiological responses of higher magnitude. These responses, however, involve complex cascade effects across many tissues and affect many physiological systems at an organismal level, some of which may entail physiological costs or trade-offs. Hence, the benefits of increasing GR abundance may be outweighed by the costs and trade-offs associated with stronger GC-mediated physiological response. These costs likely vary among environmental contexts (e.g. differing in resource availability), thus giving rise to a variety of optimal responses. Because GCs mediate changes in energy allocation across physiological systems to meet increased energetic needs [8,15,18,19,58], energy budget and resource availability may determine or shape the optimal GR phenotype in combination with the energetic challenge posed by the stressor [19]. If this is true, we could predict individuals living in environments with high resource availability to show higher GR abundance—and stronger physiological responses—as compared to individuals living in environments with low resource availability. Likewise, individuals exposed to acute, unexpected challenges (e.g. encounters with predators) would exhibit higher GR abundance since they need to mount rapid and intense GC-induced physiological responses to overcome these kinds of stimuli. We encourage upcoming research to test these predictions in a context of phenotypic adaptation in free-living species, for instance in relation to anthropogenic environmental change.

In this work we adopt a novel perspective within the study of HPA axis regulation and GC function by building a dynamic model to provide mechanistic insights on the role of GR in GC function. This model allowed us to make predictions on the expected associations among GR abundance, GC concentrations and GC-mediated physiological responses, as well as to outline the limitations of a standardized GC sampling in free-living populations exhibiting variability in GC regulatory components. We hope that our approach and predictions will increase the interest of ecologists in a more mechanistic and integrative interpretation of GC measurements in the current context of environmental change, in light of the complexity of the associations between GR, GC dynamics and phenotypic responses.

## Data Availability

The data used for model fitting was sourced from articles [29,58,60], with the authors’ permission. The R Code that implements the dynamic HPA axis model is available from the GitHub repository: https://github.com/JRubalcaba/CORT-GR_dynamics [81]. Supplementary material is available online [82].
